# Current experience and limitations of extracorporeal cardiopulmonary resuscitation for cardiac arrest in children: a single-center retrospective study

**DOI:** 10.1186/s40560-014-0068-x

**Published:** 2014-12-31

**Authors:** Kohei Tsukahara, Chiaki Toida, Takashi Muguruma

**Affiliations:** Division of Critical Care Medicine, National Medical Center for Children and Mothers, 2-10-1 Okura, 157-0074 Setagaya-ku, Tokyo Japan; Advanced emergency and critical care center, Okayama University Hospital, 2-5-1 Shikadacho, 700-8558 Kitaku, Okayama Japan

**Keywords:** Pediatric intensive care, Cardiac arrest, Extracorporeal membrane oxygenation, Extracorporeal cardiopulmonary resuscitation

## Abstract

**Background:**

There are few reports detailing the importance of extracorporeal membrane oxygenation (ECMO) for pediatric cardiac arrest in Japan. We investigated the status and issues surrounding extracorporeal cardiopulmonary resuscitation (ECPR) at our institution.

**Methods:**

Patients aged <15 years who underwent ECPR between April 1, 2003 and March 31, 2012 were eligible. The characteristics, cannulation site, durations of cardiopulmonary resuscitation (CPR), cannulation procedure, and ECMO, and neurologic outcomes were retrospectively reviewed. A favorable neurologic outcome was defined as Pediatric Cerebral Performance Categories 1 and 2.

**Results:**

A total of 21 ECPR events were identified. The median CPR and cannulation durations were 60 and 25 min, respectively. Central and peripheral access sites were employed in 15 and six cases, respectively. Five of the 21 patients (24%) were successfully weaned from ECMO and three of the 21 (14%) survived. Two of the three survivors had a favorable neurologic outcome.

**Conclusions:**

The mortality of patients undergoing ECPR at our institution was low. However, about 10% of all patients had a favorable neurologic outcome, which suggests that ECPR may be effective in pediatric cardiac arrest patients.

## Background

A return of spontaneous circulation (ROSC) following in-hospital pediatric cardiac arrests is achieved in 67% of patients; unfortunately, many patients do not survive. If conventional cardiopulmonary resuscitation (CPR) is prolonged (>30 min), the prognosis is even poorer [1].

Recently, it has been reported that the use of extracorporeal membrane oxygenation (ECMO) during CPR (ECPR) successfully resuscitated patients in cardiac arrest, with a mortality rate after ECPR of 14%–40% in adults [2,3] and 33%–51% in children [1,4–7].

In Japan, there are few reports of pediatric cases in only a few facilities. The ECPR technique is more difficult to perform in pediatric patients and requires significant resources and an experienced team. Therefore, the number of children who have been resuscitated by ECMO is unclear.

In this study, we review our experience performing pediatric ECPR and investigate the limitations that make it difficult to improve clinical outcomes.

## Methods

The National Medical Center for Children and Mothers is a 490-bed pediatric hospital with 20 beds in the pediatric ICU (PICU). This facility treats the largest number of critically ill pediatric patients in Japan, and since the hospital opened in 2002, ECMO has been performed in over 60 pediatric cases. We had 21 cases of ECPR in the same period.

### Study design and population

The medical records of pediatric patients <15 years of age who received ECPR between April 2003 and March 2012 were retrospectively reviewed for this study. The study comprised three stages as follows:

Study 1: The characteristics, cannulation site, duration of CPR, cannula insertion, and ECMO, and the neurologic outcomes were compared between surviving and non-surviving patients.

Study 2: The duration of CPR was compared before and after the introduction of the flowchart for ECMO. The cases were categorized into the pre-group (ten cases), admitted between 2003 and 2008, and the post-group (11 cases), admitted between 2009 and 2011, based on the introduction of the flowchart for ECMO.

Study 3: The duration of cannula insertion was compared according to the cannulation site.

Neurologic outcomes were determined using the Pediatric Cerebral Performance Category (PCPC) scale (1 = normal, 2 = mild disability, 3 = moderate disability, 4 = severe disability, 5 = coma or vegetative state, 6 = brain death). A favorable neurologic outcome was defined as a PCPC score of 1, 2, or no change from the initial PCPC.

### Patient characteristics

Over the 9-year study period, 21 pediatric patients underwent ECPR; their characteristics are summarized in Table 1. The patients were diagnosed as follows: 11 post-cardiosurgery cases (52%), 6 fulminant myocarditis cases, one mitral valve rupture, two congenital diaphragmatic hernia, and two unknown. The median age was 1 month (range 0 months to 13 years), and the median body weight was 4 kg (range 2–50 kg). Nine of the 11 post-cardiosurgery cases are complex congenital heart disease with single ventricle, one is hypoplastic Left Heart Syndrome and the other is aortic coarctation complex.

**Table 1:** **Characteristics of the 21 pediatric patients who underwent ECPR over the 9-year study period**

**Patient number**	**Gender**	**Disease**	**Age at admission**	**Body weight (kg)**	**The day of ECMO**	**Weaning**	**Outcome (PCPC)**	**Onset site**	**PDR**
1	F	CDH	0 month	3	5	**−**	**D**	In-hospital	9.8
2	M	Myocarditis	8 years 7 months	20	6	○	PCPC2	In-hospital	2.5
3	F	Myocarditis	3 years 11 months	14	5	**−**	**D**	Out-of-hospital	39.6
4	F	Myocarditis	7 years 6 months	27	3	**−**	**D**	Out-of-hospital	73.5
5	F	Myocarditis	1 year 7 months	10	5	**−**	**D**	In-hospital	85.0
6	M	Post-ope	1 month	5	5	○	PCPC3	In-hospital	1.7
7	F	Post-ope	0 month	3	2	**−**	**D**	In-hospital	3.5
8	F	Post-ope	0 month	3	4	**−**	**D**	In-hospital	5.9
9	M	Myocarditis	5 months	8	6	**−**	**D**	In-hospital	59.2
10	M	Post-ope	1 month	3	2	**−**	**D**	In-hospital	3.5
11	F	AHF	5 months	7	19	○	D	In-hospital	33.4
12	M	Post-ope	1 month	4	17	**−**	**D**	In-hospital	6.2
13	M	Post-ope	0 month	3	8	**−**	**D**	In-hospital	9.5
14	M	Post-ope	0 month	2	22	**−**	**D**	In-hospital	14.6
15	M	Post-ope	0 month	4	4	○	PCPC1	In-hospital	3.3
16	M	Unknown	13 years 7 months	50	6	**−**	**D**	Out-of-hospital	85.8
17	F	Post-ope	3 days	2	13	**−**	**D**	In-hospital	83.7
18	M	Post-ope	3 days	2	12	**−**	**D**	In-hospital	68.8
19	M	Post-ope	17 days	3	34	**−**	**D**	In-hospital	1.9
20	M	Myocarditis	6 years 2 months	18	8	○	PCPC6	Out-of-hospital	84.5
21	M	Unknown	2 months	6	10	**−**	**D**	In-hospital	19.8

*Abbreviations*: *CDH* congenital diaphragmatic hernia, *Post-ope* post-operative of cardiovascular surgery, *AHF* acute heart failure, *PCPC* Pediatric Cerebral Performance Category, *PDR* predictive death rate estimated by PIM2. **○** success**,** − unsuccess, *D* Death.

### Indications for ECPR

ECPR was indicated in patients in whom cardiac arrest was witnessed, those undergoing continuous CPR, those with disease or injury expected to be reversible, and those who did not respond to conventional CPR. Patients aged less than 34 gestational weeks, those weighing 1.5 kg or less, and those with severe congenital abnormality, intracranial hemorrhage, or coagulopathy were ineligible to undergo ECPR.

### Cannulation

Patients weighing ≤30 kg in body weight were cannulated through a thoracotomy site, and those weighing >30 kg were cannulated at the neck or femur. The cannula size was selected according to the body weight. In patients receiving a thoracic cannula, the cardiovascular surgeon selected the appropriate size intraoperatively.

### ECMO technique

The ECMO pump comprised a roller pump or centrifugal pump set at 100 mL/kg/min. The circuit and artificial lung were selected according to the priming volume (PV). A Biocube 2000 (PV: 239 mL, Nipro Co., Osaka, Japan) artificial lung and circuit was used in patients weighing ≤15 kg, and a Biocube 4000 (PV: 424 ml, Nipro Co.) was used in patients weighing ≥15 kg. In patients weighing <10 kg, the Excelung Kids (PV: 156 mL, Mera Co., Santa Clara, CA, USA) was used. In each circuit, a heparin coating was used to prevent coagulation, and the activated clotting time was measured at the blood removal side of the artificial lung. The heparin dose was adjusted to maintain coagulation between 180 and 200 s. The continuous hemodialysis was combined with the ECMO circuit in all cases.

### Statistical analysis

Continuous variables were evaluated with the Wilcoxon rank sum test and categorical variables with the Mann–Whitney *U* test. All tests were two-tailed, and *P* values of <0.05 were considered statistically significant. All statistical analyses were performed using STATA, version 12.1.

This protocol was approved by the Ethics Committee of the National Medical Center for Children and Mothers (approval number 961).

## Results

Cardiac arrest occurred within the hospital in 17 cases and outside the hospital in four cases. Five of the 21 patients (24%) were successfully weaned from ECMO, and three of the 21 patients (14%) survived. Furthermore, two of the three surviving patients had a favorable neurologic outcome. One patient had poor outcome, PCPC3. The causes of death in other patients were fungemia and brain death (PCPC6). Four of the out-of-hospital cardiac arrests have poor outcome. One patient is brain dead and the others are dead. Two patients had cardiac arrest during transportation.

### Study 1

The compared clinical outcomes between the survival group and non-survival group are summarized in Table 2. There were no differences between the groups in age, body weight, location of cardiac arrest, diagnosis and the duration of CPR, cannulation, and ECMO period.

**Table 2:** **Survival and none survival group**

	**None survival (** ***N*** **= 17)**	**Survival (** ***N*** **= 4)**
Age (month)	1 (0–163)^a^	1 (0–103)^a^
Male (*n*)	8 (47%)	4 (100%)
Body weight (kg)	3 (2–50)^a^	11.2 (3.5–19.7)
In-hospital event (*n*)	15 (88%)	3 (75%)
Central approach (*n*)	13 (76%)	2 (50%)
Diagnosis (*n*)		
Post-operative	9 (54%)	2 (50%)
Myocarditis	4 (23%)	2 (50%)
Others	4 (23%)	0
Daytime event (%)	10 (56%)	2 (67%)
CPR time (min)	47 (20–262)^a^	78 (60–103)^a^
Operative time (min)	17 (8–67)^a^	57 (33–84)
The duration of ECMO (day)	6 (2–34)^a^	10 (4–8)^a^

^a^Median (minimum–maximum).

### Study 2

**Figure 1:**
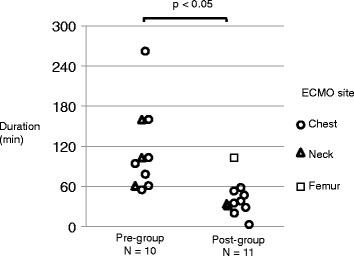
**Comparison of CPR duration before and after the introduction of the flowchart for ECPR.** The median CPR duration decreased from 86 min during 2003–2008 to 38 min during 2009–2011. The flowchart for ECMO was introduced at our facility in 2009. ECMO, extracorporeal membrane oxygenation.

The CPR duration was a median 60 min (range 20–262 min) in all cases. The CPR duration was significantly different between the pre-group and post-group (86 min [32–262 min] vs. 38 min [20–90 min], respectively) (Figure 1).

### Study 3

**Figure 2:**
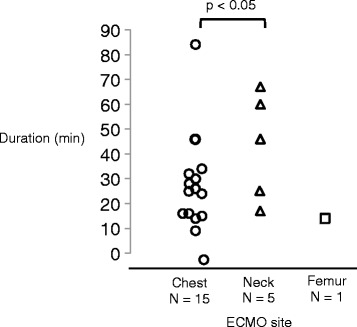
**Comparison of ECMO procedure during according to the cannulation site.** The procedure duration was shorter for ECMO cannulation using a thoracic approach than the procedures performed at the neck and femur. ECMO, extracorporeal membrane oxygenation. The chest site is indicated by the *circles*, the neck indicated by *triangles*, and the femur indicated by *squares*.

The comparison of the cannulation duration according to the cannulation site is shown in Figure 2. The procedure duration was a median 25 min (8–84 min) overall. The cannula was inserted in the chest in 15 cases (71%), the neck in five cases (24%), and the femur in one case (5%). The mean procedure duration was 24 min (8–84 min) in the chest, 56 min (17–67 min) in the neck, and 14 min in the femoral site (single case and body weight is 50 kg). The procedure duration was significantly shorter in the chest site than in the neck site.

## Discussion

In adults, the survival rate after cardiac arrest is reportedly 17%–25% for in-hospital events and 1–5% for events outside the hospital [8]. By contrast, for witnessed cardiac arrests in patients who undergo ECPR, the survival rate was 29%, which was an improvement compared with the 22% survival rate for conventional CPR, and a favorable neurological outcome occurred more frequently [9,10].

In children, the survival rate after cardiac arrest is reportedly 9%–47% for in-hospital events and 0%–29% for events outside the hospital [1,4–7]. Comparatively, the survival rate for pediatric ECPR was reportedly 33%–51% [1,4,5,7], which was higher than for conventional CPR. These reports indicate that ECPR may be more effective for pediatric cardiac arrests. However, most of these reports described cases of in-hospital cardiac arrests [1,4,5].

### ECPR indication criteria

In this study, 61% of ECPR procedures were performed following cardiac surgery, and 90% of patients had complex congenital heart disease with a low cardiac capacity and single ventricle. Chan et al. [6] reported that the CPR survival rate is low for the single ventricle form. This is attributed to the increased intra-thoracic pressure caused by chest compression, which decreases perfusion after Glenn or TCPC surgery. A long duration of chest compression may decrease the survival rate; therefore, it is important to introduce ECPR sooner in patients with a single ventricle than in patients with other diseases.

When determining indications for ECPR, it is important to distinguish cardiac arrest that occurs outside a hospital from in-hospital events. In this study, all patients who underwent ECPR following a cardiac arrest outside the hospital have poor outcome. For these particular patients, if transportation is expected to be a long duration, then ECMO during transport should be considered.

### CPR duration and quality

The median CPR duration in children is 50 min according to Morris et al. [7] and 46 min according to Alsoufi et al. [5]. Comparatively, the median CPR duration at our facility was longer at 60 min. In adults, a prolonged CPR duration reportedly decreases the survival rate [9]. Even if chest compression is performed correctly, only 20%–30% of the systolic output can be maintained [11]. Therefore, reducing the CPR duration and introducing ECMO at an earlier stage may improve the survival rate.

Despite the median CPR duration of 60 min, patient survival with a favorable neurologic outcome did occur in the present study. Alsouf et al. [5] reported that access to high-quality CPR is the most important factor for a favorable neurologic outcome. In this study, the CPR duration was very long (median 78 min [range 60–103 min]). In the two cases with favorable neurologic outcomes, CPR was performed by a bystander with witnesses, and the second occurred in a hospital with witnesses. In both cases, chest compression was conducted immediately after cardiac arrest, and a high-quality of CPR was maintained. It is important to maintain the quality of CPR.

### Shortening the duration of CPR and ECMO cannulation

The CPR duration was decreased at our facility by decreasing the duration of preparation and cannula insertion. Site preparation includes gathering the staff, preparing instruments, and assembling the circuit. Most importantly, when cannulation is performed by a cardiovascular surgeon, a smooth communication system is required. In this study, we were able to shorten the duration of CPR by using the flowchart for ECMO, first introduced in 2009. Using this system, we are able to communicate with the relevant staff and disseminate instructions to gather the necessary instruments and tools for ECMO accurately and rapidly.

Furthermore, the cannula insertion site was adjusted to reduce the procedure duration in patients weighing less than 20 kg because the procedure duration is longer at the neck, requires interruption of chest compression during insertion, and is more difficult at the neck in infants and young children. If the neck must be used, then it may be effective to use ultrasonography to evaluate the vascular lumen. It is also important to reduce the ECMO preparation duration and the interval prior to the arrival of the cardiovascular surgeon.

### Limitations

This study was performed at a single center and uses a very small sample population. Additional studies in multiple facilities are necessary.

## Conclusions

The mortality of patients undergoing ECPR at our institution was lower than that previously reported. However, 10% of patients had a favorable neurologic outcome, which suggests that ECPR may be effective for treating pediatric cardiac arrest.

To improve the clinical outcome, it is essential to determine the indication criteria for ECPR and generate a strategy to include ECMO for ECPR during transportation of children who experience cardiac arrest outside a hospital.
